# Light-Decomposable Polymeric Micelles with Hypoxia-Enhanced Phototherapeutic Efficacy for Combating Metastatic Breast Cancer

**DOI:** 10.3390/pharmaceutics14020253

**Published:** 2022-01-21

**Authors:** Yuanyuan Li, Aiyang Tong, Peiyuan Niu, Wenjing Guo, Yangye Jin, Yi Hu, Pei Tao, Wenjun Miao

**Affiliations:** School of Pharmaceutical Sciences, Nanjing Tech University, Nanjing 211816, China; liyy@njtech.edu.cn (Y.L.); 202021009050@njtech.edu.cn (A.T.); niupy@njtech.edu.cn (P.N.); wenjingguo@njtech.edu.cn (W.G.); yangyejin@njtech.edu.cn (Y.J.); huyi@njtech.edu.cn (Y.H.); 662085238012@njtech.edu.cn (P.T.)

**Keywords:** hypoxia, micelles, bioreductive prodrug, phototherapy, antitumor

## Abstract

Oxygen dependence and anabatic hypoxia are the major factors responsible for the poor outcome of photodynamic therapy (PDT) against cancer. Combining of PDT and hypoxia-activatable bioreductive therapy has achieved remarkably improved antitumor efficacy compared to single PDT modality. However, controllable release and activation of prodrug and safety profiles of nanocarrier are still challenging in the combined PDT/hypoxia-triggered bioreductive therapy. Herein, we developed a near infrared (NIR) light-decomposable nanomicelle, consisting of PEGylated cypate (pCy) and mPEG-polylactic acid (mPEG_2k_-PLA_2k_) for controllable delivery of hypoxia-activated bioreductive prodrug (tirapazamine, TPZ) (designated TPZ@pCy), for combating metastatic breast cancer via hypoxia-enhanced phototherapies. TPZ@pCy was prepared by facile nanoprecipitation method, with good colloidal stability, excellent photodynamic and photothermal potency, favorable light-decomposability and subsequent release and activation of TPZ under irradiation. In vitro experiments demonstrated that TPZ@pCy could be quickly internalized by breast cancer cells, leading to remarkable synergistic tumor cell-killing potential. Additionally, metastatic breast tumor-xenografted mice with systematic administration of TPZ@pCy showed notable tumor accumulation, promoting tumor ablation and lung metastasis inhibition with negligible toxicity upon NIR light illumination. Collectively, our study demonstrates that this versatile light-decomposable polymeric micelle with simultaneous delivery of photosensitizer and bioreductive agent could inhibit tumor growth as well as lung metastasis, representing a promising strategy for potent hypoxia-enhanced phototherapies for combating metastatic breast cancer.

## 1. Introduction

During the past decade, photodynamic therapy (PDT), with minimal invasiveness, negligible systemic toxicity, and barely intrinsic or/and acquired resistance, has been regarded as a promising alternative approach applied in clinic management of various types of cancer [1,2,3]. Photosensitizers are activated, subsequent transferring energy or electrons to surrounding molecular oxygen for generating cytotoxic reactive oxygen species (ROS), which can irreversible kill tumor cell to achieve antitumor effect [2,3,4,5]. Unfortunately, the limited solubility and stability of photosensitizers in physiological system and the hypoxic microenvironment in tumor tissue general weaken PDT efficacy [6,7]. Moreover, oxygen-consumption during treatment period may also aggravate hypoxia of tumor region, and then in turn impact the therapeutic effect and accelerate invasion and distant metastasis of tumor [8,9].

Confronting PDT-induced hypoxia issue in combating malignant tumor, several strategies have been explored, such as less oxygen dependence, oxygen co-delivery and in situ generation of oxygen during PDT [7,10,11,12]. However, these approaches have showed negligible strengthened efficacy due to heterogeneity and complicated pathological condition of tumors. Instead, the severe hypoxic microenvironment can also be utilized by introducing hypoxia-triggered bioreductive prodrugs that would be effectively activated for killing tumor cells over the course of PDT, which can act as a supplementary strategy to potentiate the antitumor efficacy [13,14,15,16,17]. Tirapazamine (TPZ), a bioreductive prodrug which is a harmless parent compound at normoxia, can generate free-radical intermediate cytotoxic species by various intracellular reductase catalytic reactions under hypoxic conditions, resulting in highly selective toxicity towards hypoxic tumor cells [14,18,19,20]. However, TPZ is poorly soluble and prone to self-aggregate at aqueous condition, greatly hampering its biomedical applications; moreover, TPZ is easily degraded in living systems, and thus a large dose of TPZ is needed in the clinic use, leading to uncertain side effects. Hence, smart vehicles that can effectively deliver hydrophobic drugs and preserve their activity are highly desirable.

To date, various types of nanoagents, which can be categorized into inorganic and organic ones, have been constructed for treating various types of cancers. For the inorganic nanocarriers, silica-based nanoparticles [21], carbon-based [22] and metal-organic framework nanomaterials [23] with unique physiochemical properties have been widely explored. In parallel, organic nanocarriers composed by naturally-occurred molecules with superior biocompatibility and biodegradability have also been intensively studied for drug delivery [24,25,26]. While inorganic nanocarriers are hardly biodegradable, raising potential toxicity concerns when applied into living system; the organic ones are unstable in physiological system, leading to burst release of drug and enhanced side effects [27,28]. In addition, nanocargos that can intelligently control drug release in response to internal stimuli (enzyme [29], redox [30], pH [31]) or external stimuli (light [22,25,32], temperature [33]) have drawn increased attention due to alleviated indiscriminate damage and enhanced antitumor efficacy of chemotherapeutics. Therefore, there has been an urgent need to develop versatile nanovehicles with good stability, safety and decomposition profile for controlled delivery of chemotherapeutics.

With these considerations in mind, we for the first time designed and fabricated an NIR decomposable polymeric micelle, designated TPZ@pCy, comprising of biocompatible PEGylated cypate (pCy) and mPEG-polylactic acid (mPEG_2k_-PLA_2k_) for controllable delivery of hypoxia-activated bioreductive prodrug (TPZ), which can combine photo/hypoxic-triggered bioreductive therapy for treatment of metastatic breast cancer. pCy, a multifunctional polymer, was synthesized according to our previous study, exhibiting photo decomposable, photodynamic and photothermal properties, which has been successfully used in malignant tumor treatments [34]. It is expected that TPZ@pCy owns the merits of remarkable colloidal stability and photonic properties, favorable light-triggered decomposition of nanocargo, and subsequent controlled release and activation of encapsulated prodrug. Furthermore, we anticipate that TPZ@pCy could preferentially accumulate in tumor tissue through the enhanced permeability and retention effect and generate abundant “heat” and ROS upon NIR irradiation followed by activation of TPZ in hypoxic microenvironment, leading to significant efficacy in suppression of tumor growth and distant metastasis. Thus, we systemically investigated the photonic performance, stability, light-decomposition profile of pCy and release behavior of TPZ. Following that, the potency of TPZ@pCy on cell entry and light-triggered antitumor effect in mouse-derived triple negative breast cancer cells were investigated. Additionally, we evaluated its biodistribution, primary tumor ablation and inhibition of distant metastasis in tumor-bearing mice.

## 2. Materials and Methods

### 2.1. Materials

Cypate was synthesized according to our previous study [26]. Methoxy-PEG_2k_-NH_2_ and methoxy-PEG-polylactic acid (mPEG_2k_-PLA_2k_) were obtained from Jinan Daigang Biotech Co., Ltd. (Jinan, Shandong, China). N,N’-dicyclohexylcarbodiimide, N-hydroxysuccinimide, 1,3-diphenylisobenzofuran (DPBF), penicillin, streptomycin, tirapazamine (TPZ), 3-(4,5-dimethylthiazol-2-yl)-2,5-diphenyltetrazolium bromide (MTT), and 4′,6-diamidino-2-phenylindole dihydrochloride (DAPI) were purchased from Sigma-Aldrich (St. Louis, MO, USA). RPMI 1640 medium, fetal bovine serum (FBS) and 24- and 96-well plates were purchased from Gibco BRL life Technologies (San Diego, CA, USA). 2,7-dichloro-dihydro-fluorescein diacetate (DCFH-DA) and reactive oxygen species assay kit were supplied by Beyotime Institute of Biotechnology, Shanghai, China. AnnexinV Alexa Fluor488/propidium iodide (PI) apoptosis assay kit was obtained from Solarbio Life Sciences Inc., Beijing, China. Anti-Ki67 (GB13030-2) and anti-caspase-3 (GB11009-1) were purchased from Wuhan Servicebio Technology Co., Ltd. (Wuhan, China). Deionized water (DW) with a resistivity higher than 18 MΩ·cm^−1^ used in the experiments was made using a Milli-Q Direct 16 Water Purification System (Bedford, MA, USA). Other chemicals were supplied by Sinopharm Chemical Reagent Co., Ltd., Shanghai, China, and used as received.

### 2.2. Cells and Animals

The mouse-derived triple-negative breast cancer cells (4T1) were obtained from the Institute of Biochemistry and Cell Biology of Chinese Academy of Sciences (Shanghai, China). 4T1 cells were cultured in RPMI Medium 1640 supplemented with FBS (10%, *v*/*v*), penicillin (100 U mL^−1^) and streptomycin (100 μg mL^−1^). The cells were maintained at 37 °C in a humidified atmosphere containing 5% CO_2_. Balb/c mice were purchased from Qinglongshan Experimental Animal Research Center, Nanjing, China. All mice were maintained in a pathogen-free environment (25 ± 2 ^o^C and 50 ± 5% humidity) on a 12 h light/dark cycle with free access to food and water throughout the experimental period. All animal experiments were conducted in compliance with Animal Care Committee of Nanjing Tech University.

### 2.3. Preparation and Characterization of TPZ@pCy

TPZ@pCy micelles were prepared by nanoprecipitation method as reported previously with slight modification [13]. In brief, TPZ, pCy, and mPEG_2k_-PLA_2k_ were dissolved in methanol, respectively, and mixed at a mass ratio of 1:1:5. The resultant mixture was vigorously stirred for 10 min at room temperature. Phosphate buffered saline (PBS, 0.15 M, pH 7.4) was added to the mixture during sonication using a bath sonicator at a frequency of 53 kHz. The remained methanol was completely removed by rotary evaporation. Free TPZ was washed out by centrifugation at 11,000× *g* for 30 min at 4 °C using an Amicon Ultra-15 centrifugal filtration device (MWCO 10K, Thermo Fisher) and TPZ@pCy above filter was re-suspended in PBS at desired concentration and stored at 4 °C in dark for future use. pCy was also prepared in similar way without TPZ.

For identification of the formed TPZ@pCy, their UV-Vis-NIR absorbance spectra were recorded over the range of 300–900 nm via Multiskan^TM^ GO microplate spectrophotometer (Thermo Fisher Scientific, Waltham, MA, USA). The hydrodynamic diameter was analyzed by dynamic light scattering (DLS) with a 10 mW He-Ne laser at 25 °C and the zeta potential value was determined by laser Doppler microelectrophoresis at an angle of 22° using a Nano ZS90 zetasizer (Malvern Instruments, Malvern, UK). The morphology and size were also observed by transmission electron microscopy (TEM) using a JEM-2100 electron microscope (JEOL, Tokyo, Japan) after stained with 1% phosphotungstic acid. The colloidal stability of TPZ@pCy in PBS and 50% FBS was monitored over 7 days.

The encapsulation efficiency (EE) and loading content (LC) of TPZ@pCy were calculated according to the absorbance of TPZ at 470 nm. Briefly, the as-prepared TPZ@pCy was centrifuged following the same experimental conditions as above and free TPZ in the supernatant was measured by a spectrophotometer. TPZ@pCy micelles were freeze-dried, and then their weight was accurately analyzed. The EE and LC were calculated by the following formulas: EE (%) = ((weight of loaded drug)/(weight of initially added drug)) × 100; LC (%) = ((weight of loaded drug)/(total weight of nanomicelles)) × 100.

### 2.4. Photonic Performance and Light-Triggered Drug Release

The photothermal property of pCy and TPZ@pCy was investigated using an IR thermal imaging system (FLIR Pro one; FLIR System Inc., Stockholm, Sweden). In short, pCy and TPZ@pCy at serial concentrations were irradiated using an 808 nm continuous wave diode laser beam (Rayan Tech., Changchun, China) at an output power of 0.48 W cm^−2^ for 10 min. The temperature of nanosuspension during laser illumination was recorded every 30 s. The photothermal heating curve of PBS was measured as a negative control. Furthermore, DPBF was used as a probe to evaluate the photodynamic capacity of pCy and TPZ@pCy. The generated ^1^O_2_ can be captured by DPBF, leading to its absorption decay at 410 nm [31]. Briefly, 2 mL oxygen-saturated dimethylformamide (DMF) containing DPBF (100 μM) and pCy or TPZ@pCy (20 μg mL^−1^, equivalent to pCy) was irradiated with 808 nm laser (0.48 W cm^−2^, 3 min). The absorption spectra were recorded by spectrophotometer. DMF containing PBS was set as a negative control.

In addition, light-triggered decomposition and drug release of TPZ@pCy were evaluated. pCy, free TPZ and TPZ@pCy (25 μg mL^−1^, equivalent to pCy) were irradiated (808 nm, 0.8 W cm^−2^) for 15 min, respectively. The absorption spectra of samples were recorded by spectrophotometer at predetermined time points. The cumulative release of TPZ from TPZ@pCy was calculated by absorbance at 470 nm according to the established calibration curve (Supplementary Materials, Figure S1). All experiments were independently performed in triplicate.

### 2.5. Cellular Uptake and Light-Triggered Intracellular ROS Generation

Next, cellular uptake behavior of TPZ@pCy was studied by fluorescence microscope and flow cytometry [34]. 4T1 cells were seeded at a density of 1 × 10^5^ cells per well into 24-well plates. When 80% confluence was reached, the cells were incubated with PBS, pCy and TPZ@pCy (20 μg mL^−1^, equivalent to pCy) for 2 h. After fixation by 4% paraformaldehyde and DAPI staining, the cells were observed under fluorescence microscope (Ti2-U, Nikon, Japan). For semi-quantitation, the cells were harvested and analyzed using a NovoCyte 2060R flow cytometer and ACEA NovoExpress software (ACEA Biosciences Inc., San Diego, CA, USA).

Meanwhile, DCFH-DA was used as a probe to quantitate the light-triggered intracellular ROS generation [35]. 4T1 cells were seeded onto 24-well plates at a density of 1 × 10^5^ cells per well. The following day, the cells were incubated with PBS, pCy and TPZ@pCy (50 μg mL^−1^ of pCy) for 2 h, and then washed with cold PBS and irradiated with 808 nm laser (0.48 W cm^−2^) for 3 min, after which the cells were incubated with fresh medium containing DCFH-DA (10 μM) for 30 min and analyzed using flow cytometer. Further, the cells were fixed with 4% paraformaldehyde and stained with DAPI, and then observed by fluorescence microscope.

### 2.6. Cytotoxicity Study

The cytotoxicity of pCy and TPZ@pCy towards 4T1 cells was evaluated by MTT cell viability assay and cell apoptosis assay [14,20]. 4T1 cells were seeded at 5 × 10^3^ cells per well in a 96-well plate and cultured under normal oxygen level (20%) for 12 h. Then, cells were treated with pCy or TPZ@pCy at serial concentrations (0–20 μg mL^−1^ of pCy, 0–110 μM of TPZ), and transferred to an incubator with either 2% or 20% oxygen atmosphere for another 24 h. For evaluation of light-triggered cytotoxicity, cells subjected to above treatment were further irradiated with 808 nm laser (0.8 W cm^−2^, 3 min) or left untreated. The cell viabilities were measured by standard MTT assay. The data were expressed as a percentage of the cell viability measured in PBS group. All groups were assessed at least in sextuplicate.

For apoptosis assay, 1 × 10^5^ cells were seeded into 24-well plates and allowed to adhere overnight. After incubation with pCy (50 μg mL^−1^), free TPZ (280 μM of TPZ), and TPZ@pCy for 2 h in dark, cells were irradiated with the 808 nm laser (0.48 W cm^−2^, 3 min) or left untreated, and then stained with 5 μL Annexin V-FITC at room temperature, followed by 5 μL PI in dark. The fluorescence intensity of the cells was analyzed by flow cytometer using green channel for Annexin V-FITC and red channel for PI, respectively.

### 2.7. Biodistribution In Vivo

The whole-body fluorescence imaging system was used to confirm the biodistribution of TPZ@pCy in tumor-bearing mice. In brief, Balb/c mice were randomly divided into four groups (*n* = 3) and subcutaneously inoculated with 5 × 10^5^ 4T1 cells into dorsal right side and tumors were allowed to become established over time. When the tumor volume reached approximately 80 mm^3^, the mice were intravenously administered free cypate (5 mg kg^−1^ of cypate), pCy and TPZ@pCy (5 mg kg^−1^ of TPZ), respectively. At predetermined time points post-administration, the cypate fluorescence signal was assessed using an IVIS Lumina imaging system (Ex-745 nm; Em-800 nm, Spectrum BL, Boston, MA, USA). The mice were sacrificed at 48 h post-administration. Tumor and major organs (heart, liver, spleen, lung and kidney) were excised for ex vivo imaging.

### 2.8. Antitumor Efficacy of Multiple Therapy In Vivo

Therapeutic effect of TPZ@pCy in vivo was evaluated in 4T1 tumor-bearing mice. Tumor-bearing mice were established and treatment commenced when the average tumor volume was around 50 mm^3^. The mice were randomly divided into six groups (*n* = 6) and intratumorally injected with PBS, PBS+light, TPZ@pCy, free TPZ, pCy+light and TPZ@pCy+light (1 mg kg^−1^ of TPZ) every three days, respectively. At 1 h after injection, the mice were irradiated with 808 nm laser (0.4 W cm^−2^) for 5 min or left untreated. Light-induced temperature changes in the tumor site were recorded using a real-time IR thermal imaging system. In addition, the tumor volume was monitored using a digital caliper every two days. All tumor-bearing mice were photographed at day 1, 6, 12 and 18 post-treatments, and then sacrificed at day 18 and the tumors were collected, photographed, weighed and used for hematoxylin and eosin (H&E), Ki67 and Caspase-3 immunofluorescence staining according to standard experimental protocol.

### 2.9. Anti-Metastasis Potency In Vivo

To explore the inhibition of metastasis potential in vivo, the 4T1 tumor-bearing mice subjected to TPZ@pCy treatment as described in Section 2.8 were sacrificed at day 18 post treatment. Lung tissues were excised and washed with cold PBS, and then photographed and the number of metastatic modules in lung was counted to evaluate the inhibition capability of distant metastasis. In parallel, lung tissues were fixed with 4% paraformaldehyde, embedded in paraffin, sliced, and stained with H&E for histopathological analyses.

### 2.10. Biosafety Study In Vivo

For evaluating the biosafety profile of TPZ@pCy in vivo, the 4T1 tumor-bearing mice were treated with TPZ@pCy as described in Section 2.8. The body weight of mice was monitored every two days throughout the experimental period. At 18 days post administration, all mice were sacrificed and major organs (heart, liver, spleen, and kidney) were collected and the following procedures were same as above for histopathological analysis.

### 2.11. Statistics Analysis

Origin 8.0 was used for all statistical analyses. ANOVA was used to analyze all data with a Student-Newman-Keuls test for post-hoc pairwise comparisons. Differences with *p*-values < 0.05 were considered statistically significant.

## 3. Results and Discussion

### 3.1. Preparation and Characterization of TPZ@pCy

**Figure 1:**
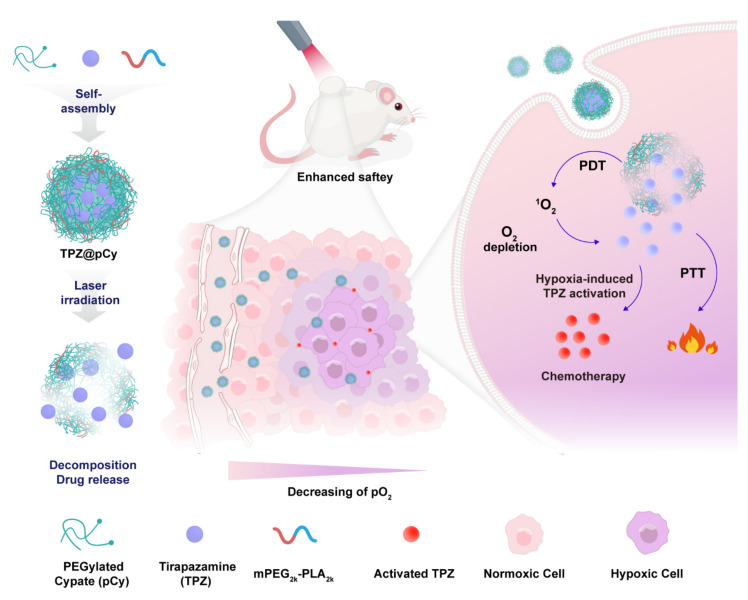
Schematic illustration of the fabrication process of TPZ@pCy and its mechanism of action for achieving controllable decomposition of nanocargo and delivery and activation of tirapazamine.

**Figure 2:**
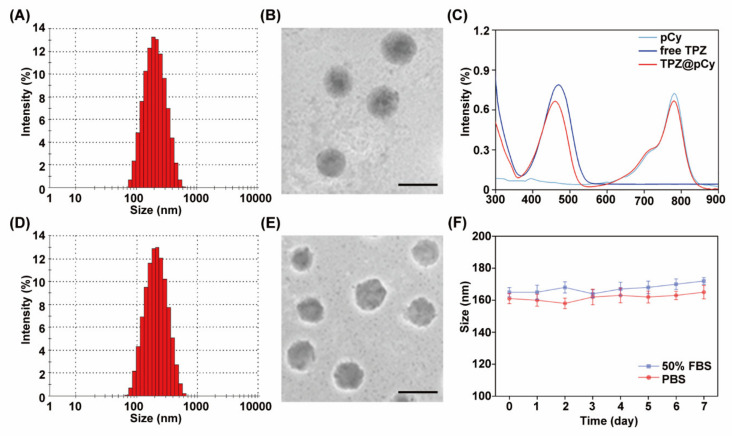
Characterization of pCy and TPZ@pCy. Hydrodynamic diameter of pCy (**A**) and TPZ@pCy (**D**) was measured by dynamic light scattering. TEM images of pCy (**B**) and TPZ@pCy (**E**). Scale bar: 200 nm. (**C**) UV-Vis-NIR spectra of pCy, free TPZ, and TPZ@pCy were acquired by a microplate spectrophotometer. (**F**) Colloidal stability of TPZ@pCy in PBS and 50% FBS, respectively. Data are shown as means ± S.D. (*n* = 3).

The preparation of TPZ@pCy is schematically illustrated in Figure 1. First, PEGylated cypate (pCy) was synthesized by conjugating cypate with mPEG_2k_-NH_2_ via amide bond [32], obtaining a dark greenish powder. pCy can spontaneously self-assemble into nanomicelle in which the hydrophobic cypate constitutes the core and the hydrophilic PEG faces towards water in an aqueous condition. Following that, TPZ, a hydrophobic bioreductive prodrug, was encapsulated in the nanomicelle, driven by the hydrophobic interaction between TPZ and cypate to get TPZ@pCy, and then mPEG_2k_-PLA_2k_ was used for enhancing its colloidal stability and drug encapsulation efficiency. DLS analysis showed that pCy and TPZ@pCy were monodispersed with mean hydrodynamic sizes of 162.6 ± 5.7 nm and 165.1 ± 4.9 nm and a polydispersity index of 0.15 and 0.19, respectively (Figure 2A,D). As revealed by TEM images, both pCy and TPZ@pCy have a uniform spherical morphology with average size of approximately 126 nm (Figure 2B,E). The ζ potential values were found to be −0.51 ± 0.20 mV for pCy and −0.45 ± 0.31 mV for TPZ@pCy, which reveals that both of them are close to neutral without notable difference. We confirmed that the loading of TPZ has no significant impact on their size, morphology and ζ potential.

As shown in Figure 2C, TPZ@pCy exhibited intense absorption in visible and NIR region, with characteristic peaks at 460 and 790 nm, characteristic of free TPZ and cypate, respectively. The DL and EE were calculated to be 6.3% and 44.2%, respectively. We observed negligible appearance changes of TPZ@pCy in different types of media and its hydrodynamic size in PBS or 50% FBS exhibited no notable change over 7 days (Figure 2F). Collectively, we successfully fabricated TPZ@pCy with satisfactory colloidal stability.

### 3.2. Light-Triggered Heat and Singlet Oxygen Generation

**Figure 3:**
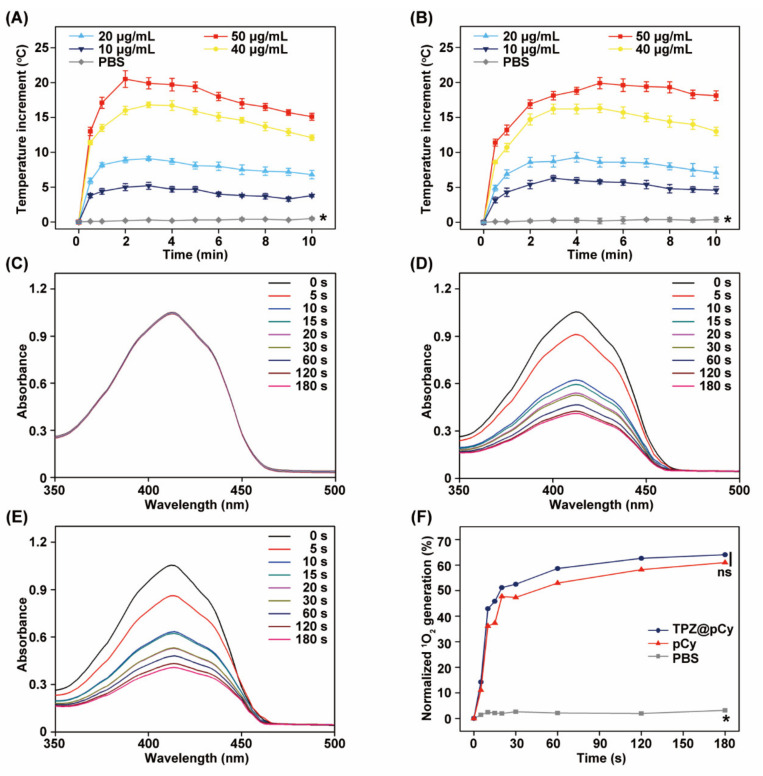
Photothermal and photodynamic performance of TPZ@pCy. Photothermal effects of pCy (**A**) and TPZ@pCy (**B**) with serial concentrations upon 808 nm laser irradiation (0.48 W cm^−2^). Free TPZ (**C**), pCy (**D**), or TPZ@pCy (**E**) was irradiated with 808 nm laser (0.48 W cm^−2^, 3 min) in the presence of DPBF (100 μM) and their UV-vis spectra were monitored at predetermined time points. (**F**) The absorbance reduction at 410 nm indicates the generation of singlet oxygen, which was quantitated and compared among groups. Data are shown as means ± S.D. (*n* = 3). * *p* < 0.05, ns: no significance.

We next explored the photothermal behavior of TPZ@pCy in response to 808 nm laser irradiation using an IR thermal camera in real time. Compared with PBS, the temperature of pCy and TPZ@pCy was remarkably enhanced upon illumination at low intensity (0.48 W cm^−2^), demonstrating that both of them have an excellent photothermal performance. Of note, we found that their temperature increase was proportional to the irradiation time and concentration of pCy. After irradiation for 10 min, the temperature of pCy and TPZ@pCy increased by 16.7 and 17.9 °C at 50 μg mL^−1^, respectively (Figure 3A,B). Furthermore, similar photothermal profile of pCy and TPZ@pCy with serial concentrations was observed, suggesting a negligible impact of TPZ loading on photothermal potency of TPZ@pCy. We also observed that the temperature of pCy and TPZ@pCy rapidly rose and reached plateau within 2 min and then slightly declined with prolonging the irradiation time. This may be explained by light-triggered degradation of nanocarrier (pCy), in agreement with our previous studies [33].

Figure 3C–F showed the singlet oxygen (^1^O_2_) generation ability of TPZ@pCy. Barely decay of DPBF absorption at 410 nm in free TPZ group was observed under 808 nm laser irradiation within 180 s, suggesting that free TPZ did not have any photosensitizing effect. However, TPZ@pCy exhibited a comparable DPBF-consumption rate to pCy, implying their similar ^1^O_2_ producing potential upon laser irradiation (Figure 3D,E). After irradiation for 180 s, the ^1^O_2_ generation percentage of pCy and TPZ@pCy was achieved to 68.3% and 69.5%, respectively (Figure 3F). Moreover, the encapsulation of TPZ did not compromise the photosensitizing activity of pCy, which was also observed in our previous studies on indocyanine green or porphyrins analog-based nanosystem [36,37]. Taken together, TPZ@pCy exhibited remarkable photothermal and photodynamic performance in response to 808 nm light irradiation.

### 3.3. Light-Induced Decomposition and Drug Release

**Figure 4:**
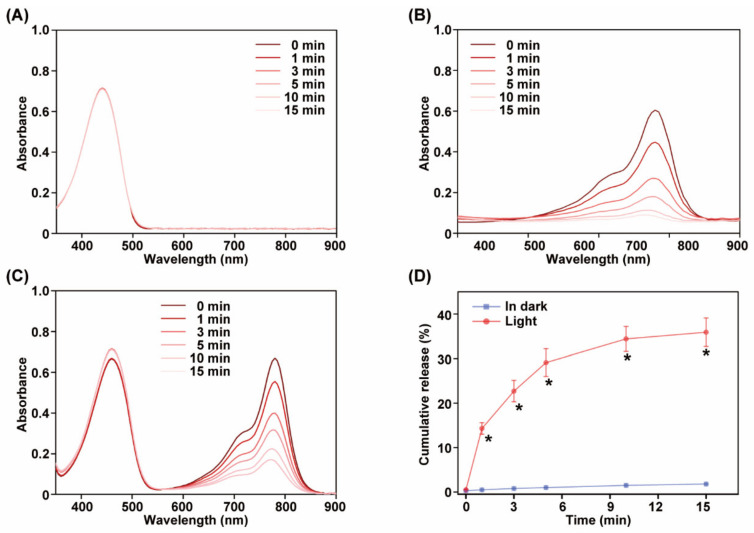
Light-triggered decomposition and drug release of TPZ@pCy. Change of Vis-NIR spectra of free TPZ (**A**), pCy (**B**) and TPZ@pCy (**C**) upon 808 nm laser irradiation (0.8 W cm^−2^) over time. (**D**) Drug release profiles of TPZ@pCy over irradiation course. Data are shown as means ± S.D. (*n* = 3), * *p* < 0.05.

For validation its light-triggered decomposition ability, TPZ@pCy was irradiated with 808 nm laser at 0.8 W cm^−2^ for 15 min and its visible spectrum was recorded using a spectrophotometer at predetermined time points. As shown in Figure 4B,C, compared with free TPZ, the absorption at 790 nm was significantly decreased during irradiation in pCy and TPZ@pCy groups. At 15 min post-illumination, only 9.5% and 11.4% of the characteristic absorbance for pCy and TPZ@pCy remained, respectively, indicating fast light-triggered decomposition. This phenomenon may be attributed to the light-triggered destruction of Cy chromophore structure and subsequent degradation [32], which also explained the fact that the attenuated photothermal potency with prolonging irradiation time. Otherwise, the influence of TPZ on the light-induced decomposition profile of TPZ@pCy was scarcely observed. Meanwhile, the absorption peak at 470 nm has no significant change during illumination in free TPZ and TPZ@pCy group, suggesting superior photostability of TPZ (Figure 4A). Thus, pCy-based nanosystem is light decomposable, which might be used as smart drug delivery nanocarrier.

In in vitro release test, TPZ@pCy was dispersed in PBS (pH 7.4) at 37 °C with illumination or in dark. It was found that only a tiny proportion of TPZ was liberated from TPZ@pCy in dark (Figure 4D), which can be ascribe to the good colloidal stability of pCy-based nanomicelle. By contrast, the cumulative released TPZ was remarkably increased, reaching 37.3% (versus 2.7% in dark) after irradiation for 15 min, displaying an obvious light-responsive drug release capacity of TPZ@pCy. Collectively, TPZ@pCy is suitable for TPZ delivery with a well-controllable release pattern, accompanied by the decomposition of nanovehicle (pCy), which is likely to provide better safety profile in vivo.

### 3.4. Efficient Cellular Uptake and Light-Triggered Intracellular ROS Generation

**Figure 5:**
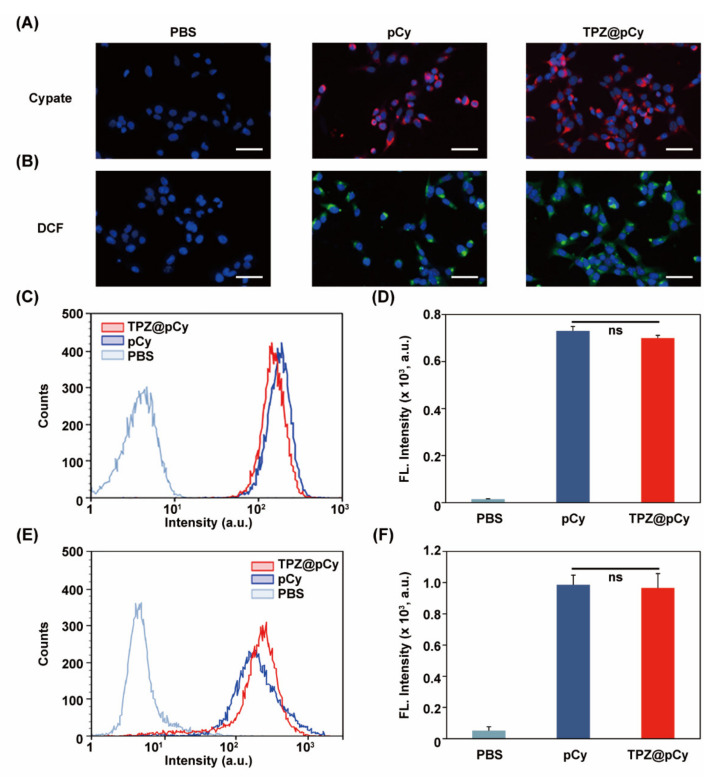
Cellular uptake behavior and intracellular ROS generation of TPZ@pCy in vitro. Fluorescence images (**A**) and flow cytometry analysis (**C**) of cellular uptake of 4T1 cells treated with PBS, pCy, or TPZ@pCy (20 μg mL^−1^ of Cy) for 2 h. (**D**) Plot showing the fluorescence intensity based on (**C**). Fluorescence images (**B**) and flow cytometry analysis (**E**) of intracellular ROS generation of 4T1 cells that received various treatments as indicated and exposed to 808 nm laser, and then intracellular ROS was detected with DCFH-DA (green) and cell nuclei was stained with DAPI (blue). (**F**) Plot showing the fluorescence intensity based on (**E**). Scale bar: 50 μm. Data are shown as means ± S.D. (*n* = 3), ns: no significance.

Fluorescence signal of cypate was used to track the uptake behavior of TPZ@pCy into 4T1 cells. We found that evident red fluorescence appeared in the cytosol of most cells treated with either pCy or TPZ@pCy (Figure 5A and Supplementary Materials, Figure S2). Semi-quantitative analysis revealed that the mean fluorescence intensities of cells treated with pCy or TPZ@pCy were similar without statistical differences (Figure 5C,D). These results demonstrated the effective internalization of TPZ@pCy into cancerous cells, which may improve the eventual performance in light-mediated multiple therapies.

Furthermore, the level of intercellular ROS generation in TPZ@pCy-treated 4T1 cells was investigated. When cells were treated with pCy or TPZ@pCy in dark, negligible green fluorescence was detected (Supplementary Materials, Figure S3). Conversely, bright green fluorescence (DCF) was observed in the cells treated with TPZ@pCy plus light (808 nm, 0.48 W cm^−2^, 3 min), suggesting largely intracellular ROS generation (Figure 5B and Supplementary Materials, Figure S4). Flow cytometry analysis (Figure 5E,F) also revealed that the fluorescence intensity of 4T1 cells treated with pCy was barely different from those treated with TPZ@pCy, indicating their similar ROS generation potency with irradiation. Therefore, TPZ@pCy can readily enter cells and generate a large quantity of ROS in response to laser irradiation, which is expected to exacerbate hypoxia microenvironment and activate bioreductive prodrug (TPZ), enlarging the synergetic anticancer effect.

### 3.5. Antitumor Efficacy In Vitro

**Figure 6:**
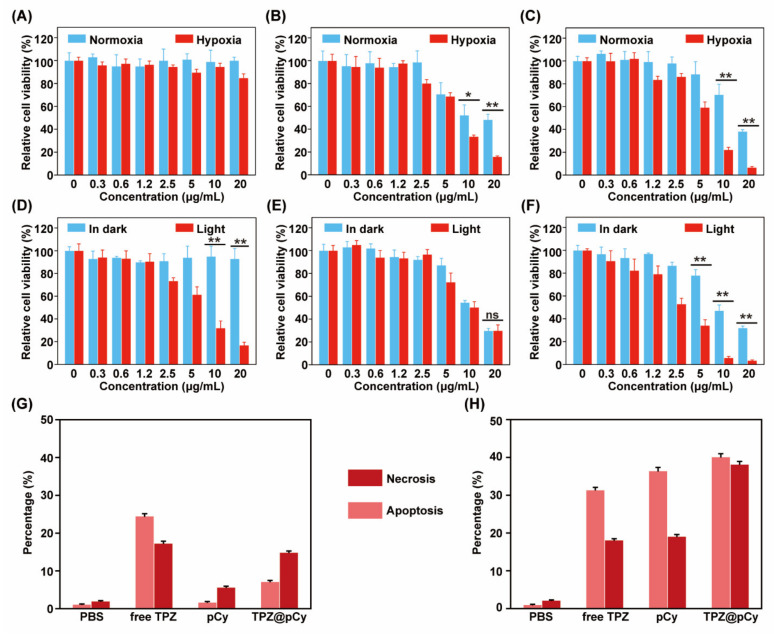
Cytotoxicity of TPZ@pCy in vitro. Relative cell viability of 4T1 cells treated with pCy (**A**), free TPZ (**B**) or TPZ@pCy (**C**) with a series concentration in normoxia and hypoxia for 24 h, respectively. Relative cell viability of 4T1 incubated with pCy (**D**), free TPZ (**E**) or TPZ@pCy (**F**) for 10 h and then irradiated with 808 nm laser (0.8 Wcm^−2^, 3 min) or left untreated. 4T1 cells incubated with pCy (50 μg mL^−1^), free TPZ or TPZ@pCy (50 μg mL^−1^ of TPZ) for 2 h and then irradiated with 808 nm (0.48 W cm^−2^, 3 min) or not, using the Annexin V-FITC/PI staining. The percentage of 4T1 cells that underwent apoptosis and necrosis in dark (**G**) or plus light (**H**) after treatments. Data are shown as mean ± S.D. (*n* = 3). * *p* < 0.05, ** *p* < 0.01, ns: no significance.

To assess the oxygen content-dependence of cytotoxicity, normoxia (20% of oxygen) and hypoxia (2% of oxygen) environments were used. We found that the pCy-treated cells in dark remained over 97% of viability, implying a satisfied cytocompatibility of pCy (Figure 6A). Comparatively, both of free TPZ and TPZ@pCy showed remarkable dose-dependent toxicity profiles under hypoxic condition and much stronger than that under normoxic condition, attributing to hypoxia-triggered antitumor activity of TPZ. More than 80% of cells treated with free TPZ and TPZ@pCy were killed in hypoxia when the concentration of TPZ reached 110 μM (Figure 6B,C). Upon 808 nm laser irradiation, the cell viability of free TPZ group exhibited negligible difference compared to that in dark (Figure 6E). In sharp contrast, only 32.1 ± 6.2% of cells incubated with pCy at 10 μg mL^−1^ following irradiation for 3 min was survived, displaying a notable photodynamic antitumor effect of pCy, in accordance with the results of intercellular ROS generation (Figure 6D). It is worthwhile to note that TPZ@pCy plus light exhibited excellent cell killing activity in hypoxia and the cell viability at 20 μg mL^−1^ was much lower than that of pCy under identical experiment condition (3.5 ± 0.5% for TPZ@pCy versus 16.8 ± 2.7% for pCy) (Figure 6F), implying great antitumor potential of TPZ@pCy in vitro.

Annexin-V/PI staining and flow cytometry analysis were further employed for semi-quantitative assessing the apoptotic cells subjected to various treatments as above. The percentage of live cells in TPZ group with irradiation or not decreased to 57.8% and 50.3%, respectively, showing a similar ability to induce cell apoptosis. Meanwhile, early apoptosis and late apoptosis/necrosis in pCy group plus light increased to 36.4% and 19.1%, respectively (Figure 6G,H). These results demonstrated that neither TPZ nor pCy plus light could efficiently kill cancerous cells. While the potential of TPZ@pCy upon irradiation to induce apoptosis was notably enhanced, with 40.1% and 38.2% of cells in early apoptosis and late apoptosis/necrosis, respectively (Supplementary Materials, Figure S5), which again verified that TPZ@pCy can rapidly release a large amount of heat and ROS under irradiation and subsequently activate TPZ in severe hypoxia microenvironment to accelerate apoptosis/necrosis. Collectively, TPZ@pCy displays a remarkable antitumor performance via synergetic photo/hypoxia-activated bioreductive therapy.

### 3.6. Biodistribution of TPZ@pCy In Vivo

**Figure 7:**
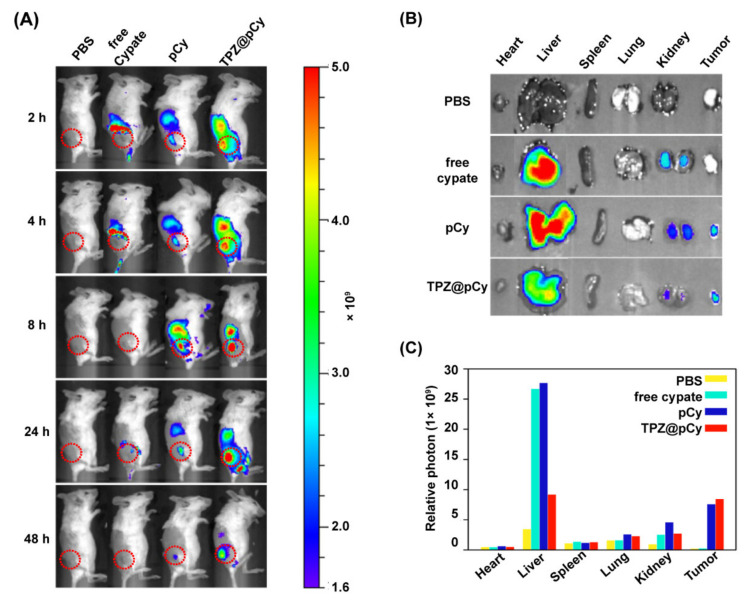
In vivo distribution of TPZ@pCy. (**A**) Whole body fluorescence images of 4T1-bearing mice intravenously received PBS, free cypate (5 mg kg^−1^), pCy, or TPZ@pCy (5 mg kg^−1^ of TPZ). (**B**) Ex vivo fluorescence images of major organs and tumors at 48 h with treatment. (**C**) Plot showing photon counts based on (**B**). Red circles denote tissue region. Data are shown as means ± S.D. (*n* = 3).

Next, we continued to investigate the biodistribution of TPZ@pCy in vivo using an IVIS Lumina imaging system. Figure 7A showed the fluorescence images of 4T1 tumor-bearing mice received various treatments. It was found that the fluorescence signal in the tumor of mice treated with free cypate was weak and quickly decayed, possibly due to fast elimination of free cypate from body. By contrast, intensive fluorescence was observed at tumor tissue and other organs of mice both in pCy and TPZ@pCy groups at 2 h post-injection, and maximized at 8 h, indicating that pCy and TPZ@pCy can effectively accumulate in tumor sites. For the ex vivo biodistribution imaging, the mice were sacrificed at 48 h post-treatment and the tumor and major organs were collected for assessment. It was found that pCy and TPZ@pCy were highly accumulated in tumor tissue and their fluorescence intensity was much higher than that in free cypate group. Furthermore, stronger fluorescence in liver and kidney was observed in free cypate and pCy group when compared to TPZ@pCy group (Figure 7B,C). Therefore, we preliminarily conclude that TPZ@pCy can effectively accumulate in tumor sites, which is greatly advantageous for light-mediated multiple-therapy against metastatic breast cancer.

### 3.7. Therapeutic Efficacy In Vivo

**Figure 8:**
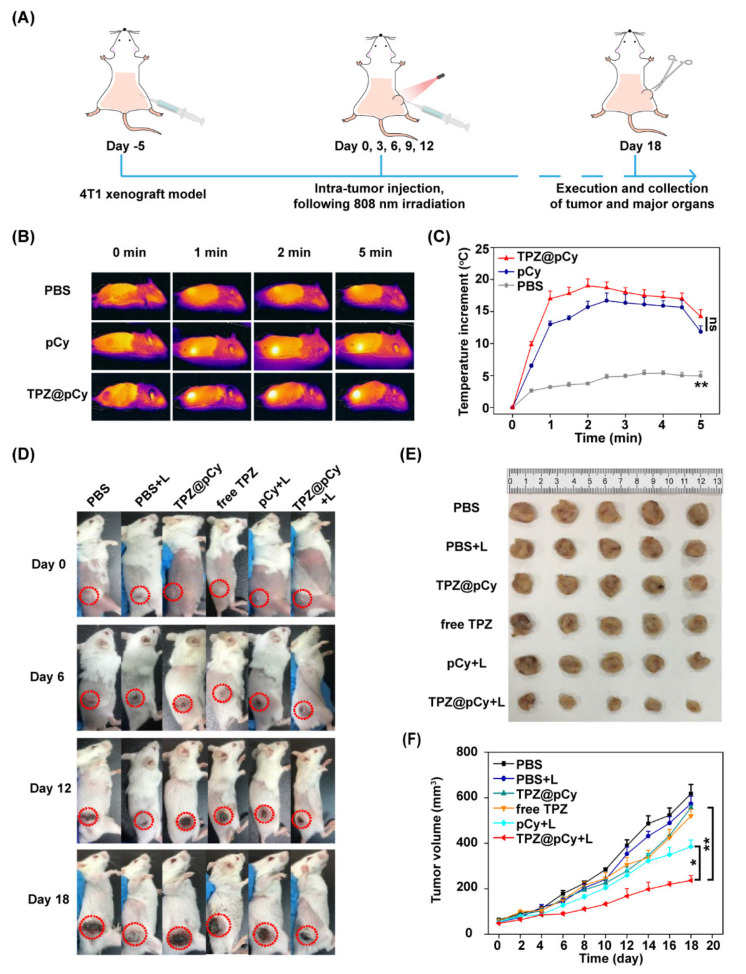
Light-induced antitumor effect of TPZ@pCy in vivo. (**A**) Schematic illustration for the establishment of in vivo study. (**B**) Real time thermal images of 4T1 tumor-bearing mice intravenously injected with PBS, pCy or TPZ@pCy (1 mg kg^−1^, equivalent to pCy) and exposed to 808 nm laser (0.4 Wcm^−2^, 5 min) at 1 h post-injection. (**C**) Photothermal heating curves of tumor region of mice subjected to various treatments. (**D**) The appearances of tumor sites were indicated by red circles. (**E**) Photograph of tumors excised from mice in all groups. (**F**) Tumor growth profile of 4T1 tumor-bearing tumor mice with indicated treatments. Data are shown as mean ± S.D. (*n* = 6). * *p* < 0.05, ** *p* < 0.01, ns: no significance.

Figure 8A exhibits the experiment scheme of animal study from tumor model construction to evaluation of therapeutic efficacy. We first explored the photothermal profile of TPZ@pCy in 4T1 xenograft-tumor bearing mice. As shown in Figure 8B, PBS-treated mice barely showed temperature elevation in tumor region. However, the temperature of tumor area of mice in pCy and TPZ@pCy group rapidly increased within 5 min, reaching up to 49 °C and 52 °C, respectively (Figure 8C). It is expected that the superior photothermal effect of TPZ@pCy is advantageous of irreversibly killing cancer cells and tumor ablation.

The photographs of mice received various treatments at predetermined time points showed that the tumor of mice had snowballed in PBS group plus light or left untreated and negligible inhibitory effect in both of them were observed, suggesting harmlessness of NIR laser irradiation (Figure 8D,E). Meanwhile, we observed marginal antitumor effect of free TPZ or TPZ@pCy without irradiation and their tumor growth was scarcely suppressed with a similar trend of the volume and weight change of tumor tissue. However, the tumor inhibition rate in pCy plus laser group was averagely 46.9%, implying that pCy-mediated phototherapy could partially retard tumor progression. Of special note, the tumor inhibition rate of TPZ@pCy upon irradiation was 70.4%, greatest among all groups (Figure 8F and Supplementary Materials, Figure S6). This can be partially explained by the fact that TPZ@pCy generated hyperthermia and ROS for phototherapy over course of the irradiation and in turn aggravated hypoxia in tumor region, which further can activate TPZ for bioreductive therapy, achieving a significant synergistic antitumor efficacy.

**Figure 9:**
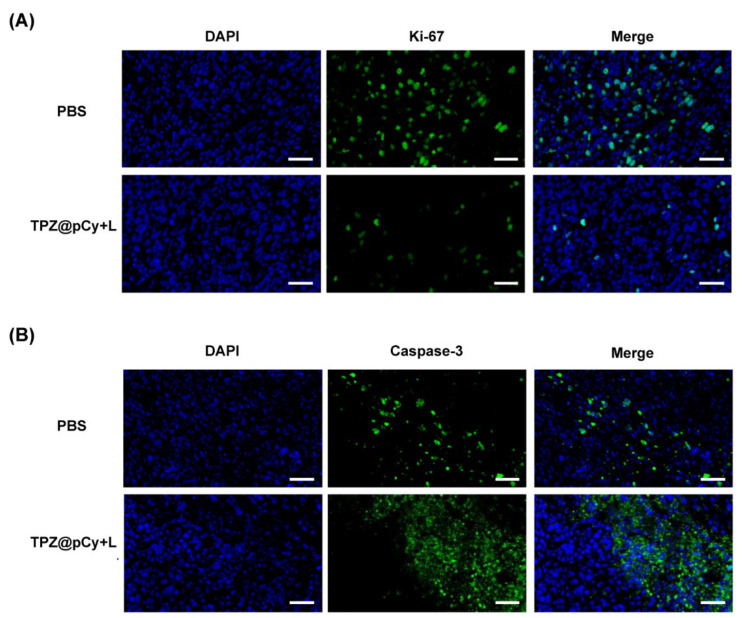
Immunofluorescent staining of tumor tissue after treatments. Immunofluorescent images (green) showing the expression of Ki67 (**A**) and Caspase-3 (**B**) in tumor region of mice received PBS or TPZ@pCy plus light treatment. Cell nuclear was stained by DAPI (blue). Scale bar: 100 μm.

We further confirmed the antitumor potency of TPZ@pCy via pathological analysis, including immunofluorescence and H&E staining assays. Ki-67 antigen and caspase-3 was used to evaluate the extent of tumor cell proliferation and apoptosis, respectively. Obviously, the expression of Ki-67 in tumor tissue in TPZ@pCy group plus light was notably decreased and much lower than that in PBS group, implying a remarkable retarded tumor cell proliferation after treatment (Figure 9A). While the tumor tissue exhibited increased caspase-3 expression at identical condition, suggesting markedly accelerated cell apoptosis (Figure 9B). Additionally, H&E staining analysis showed the infiltration of tumor cells with large nuclei and abundant chromatin of tumor tissue in PBS plus light or not and free TPZ groups. By contrast, we found that massive collapse of cell nuclei and cytosol degradation in TPZ@pCy plus light-treated tumor. Otherwise, only a tiny proportion of sporadic necrotic cells were found in pCy plus light group (Supplementary Materials, Figure S8). Thus, these phenomena again proved the excellent antitumor performance of TPZ@pCy over irradiation course.

### 3.8. Anti-Metastasis Potency In Vivo

**Figure 10:**
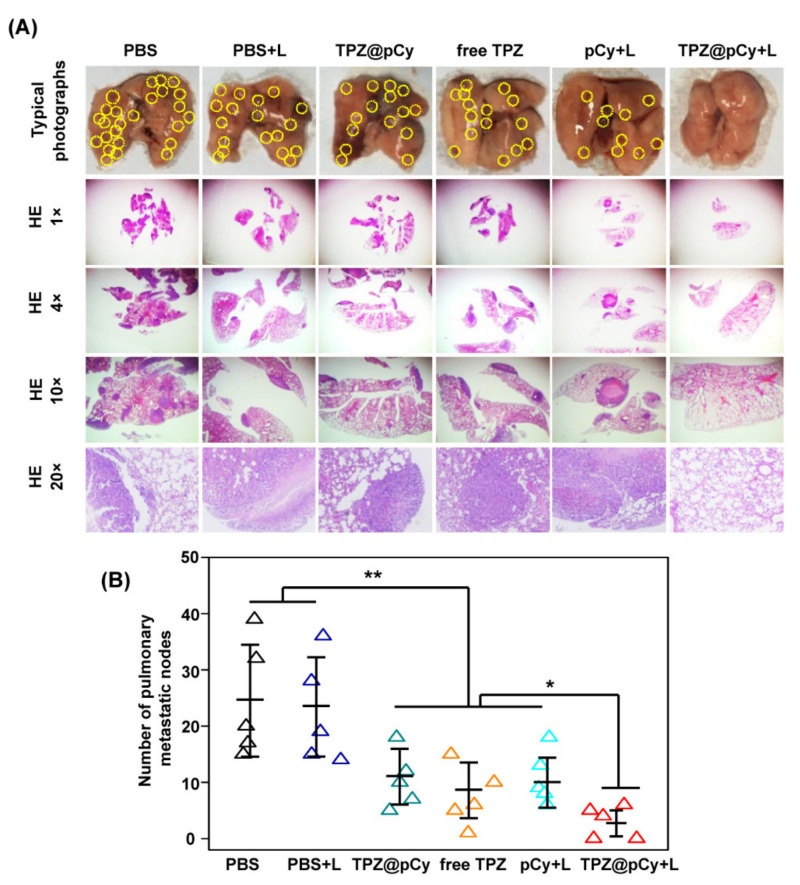
Anti-metastatic capacity of TPZ@pCy in vivo. (**A**) Representative pictures of whole lungs collected from 4T1 tumor-bearing mice receiving different treatments as indicated and H&E-stained of lung tissue sections (1×, 4×, 10×, 20×). (**B**) Number of pulmonary metastatic nodules of 4T1 tumor-bearing mice after various treatments. Yellow circle denotes surface lung metastases. Data are shown as mean ± S.D. (*n* = 6). * *p* < 0.05, ** *p* < 0.01.

To investigate the anti-metastasis activity of TPZ@pCy, the mice were euthanized at day 18 post-treatment and lung was collected for counting metastases nodes and histological analysis. As shown in Figure 10A, we found large number of metastatic nodes in PBS group with irradiation or not, implying failure of suppression of distant pulmonary metastasis of phototherapy regimen alone. Otherwise, the lung metastasis of mice treated with free TPZ or TPZ@pCy alone was marginally decreased by 48.3% and 51.7%, respectively, which was comparable to that of mice in pCy group with irradiation. This fact demonstrated that photodynamic or hypoxia-induced bioreductive therapy alone has slightly inhibitory anti-metastasis ability. Conversely, it was found that TPZ@pCy plus light showed notable suppression capacity of distant pulmonary metastasis and its lung metastasis rate was significantly decreased by ~90% (Figure 10B). Therefore, TPZ@pCy not only remarkably inhibited primary tumor growth but also effectively suppressed the distant lung metastasis via hypoxia-enhanced phototherapies.

Considering their similarities to orthotopic tumor models, including tumor progression, histopathological characteristics, and lung metastasis profile [14,15,16,38,39,40], subcutaneously xenograft breast cancer model was used in our study for evaluation of lung metastasis suppression. We observed the potent efficacy of TPZ@pCy on lung metastasis suppression in 4T1 tumor-bearing mice. Whereas, it is worth to note that orthotopic breast cancer model shows increased malignant behavior, such as microvessel density and lung metastasis, compared with that of subcutaneous breast cancer model, which is closer to real pathology of patient in clinic [38]. For the translation into clinic use, the anti-metastasis potency of TPZ@pCy in orthotopic breast cancer model should be explored in future.

### 3.9. Safety Assessment

The biosafety of TPZ@pCy was preliminarily evaluated by the changes of body weight and histological analysis of major organs. The weight of mice showed no noticeable loss in each group throughout treatment periods (Supplementary Materials, Figure S7). Post-treatment, heart, liver, spleen, and kidney were collected for histopathological analysis and negligible tissue lesion and physiological morphology change of major organs was observed (Supplementary Materials, Figure S8). Thanks to the bioreductive characteristics of TPZ, we observed negligible toxicity of both free TPZ and TPZ@pCy, which is consistent with other published results of TPZ delivery system, such as light/hypoxia-activated nanodrug [32] and stimuli-responsive covalent organic framework nanoparticle [41]. Again, the biocompatibility of pCy and TPZ@pCy further verified the good safety records of PEGylated cypate and PEG-PLA [34,42].

Recently, cypate has gain received increased interest in optical imaging and multiple phototherapies against malignant tumors due to its excellent optical properties. Beyond as a fluorescent probe for tumor diagnosis [43,44,45], cypate-based phototherapies can be combined other treatment modality, like gene therapy and chemotherapy, to achieve synergistic antitumor effect. Wang et al. reported that loading cypate-conjugated upconversion nanoparticle (UCNP) with a small interfering RNA gene against heat shock protein 70 (UCNP-cy-siRNA) enhances the damage towards tumor cells [46]. However, the heavy metal nature of UCNP might inevitably induce long-time cumulative toxicity in living system [47]. Comparatively, biocompatible polymers (PEG, PLA) used in our current study could minimize the toxicity concern. Besides, chemotherapeutics (doxorubicin and paclitaxel, etc.) are also reported to co-encapsulated with cypate into nanocargo for synergistic therapy [48,49,50,51,52]. Even enhanced tumor accumulation of toxic chemotherapeutics can be realized by nanomedicine, their distribution in non-target sites cannot be ignored. TPZ, as a bioreductive agent used in our study, undergoes conversion into active form in hypoxic environments, which mostly occurred in solid tumors. This mode of action would further attenuate the toxicity of chemotherapy. Collectively, TPZ@pCy, light-decomposable nanomicelle-based photo/bioreductive therapy against breast cancer showed favorable inhibition of tumor growth and distant lung metastasis with an acceptable safety profile in mice.

## 4. Conclusions

In summary, we have successful development of a versatile near infrared light-decomposable polymeric micelle, TPZ@pCy, which can simultaneously deliver photosensitizer and bioreductive drug for effective light-triggered multiple therapy in combating metastatic breast cancer. TPZ@pCy showed superior colloidal stability in different media, excellent photodynamic and photothermal potential, light-triggered drug release and decomposition of nanocarrier simultaneously upon NIR laser irradiation, leading to a great advantage of bioreductive prodrug activation and notable potency of killing 4T1 cells in vitro. Furthermore, we also demonstrated TPZ@pCy could preferentially accumulate in tumor region in mice, providing an opportunity for accelerated primary tumor ablation and significant suppression of distant pulmonary metastasis with acceptable safety in vivo. Taken together, with great stability, safety, light-triggered nanocargo decomposition and drug release, remarkable photonic potency, and therapeutic efficacy, TPZ@pCy, a multifunction polymeric micelle, is believed to be developed into an intelligent nanomedicine combining photo/bioreductive therapy for metastatic breast cancer treatment, with great promise for translation into clinical practice in the future.

## Data Availability

The data presented in this study are available on request from the corresponding author.

## Supplementary Materials

The following are available online at https://www.mdpi.com/article/10.3390/pharmaceutics14020253/s1, Figure S1. The calibration curve of TPZ in methanol, concentration range: 0.5–10 μg mL^−1^. Figure S2. Fluorescence images of 4T1 cells treated with PBS, pCy or TPZ@pCy (20 μg mL^−1^, equivalent to pCy) for 2 h, respectively. Cell nuclei was stained with DAPI (blue). Scale bar: 50 μm. Figure S3. Fluorescence images of 4T1 cells incubated with PBS, pCy or TPZ@pCy (50 μg mL^−1^ of pCy) for 2 h in dark. (A) Intracellular ROS generation was detected with DCFH-DA (green) and cell nuclei was stained with DAPI (blue). Scale bar: 50 μm. (B) Flow cytometry analysis of 4T1 cells treated as identical conditions as above. (C) Plot showing the fluorescence intensity corresponding to (B). Figure S4. Fluorescence images of 4T1 cells incubated with PBS, pCy or TPZ@pCy (50 μg mL^−1^ of pCy) for 2 h and irradiated with 808 nm laser (0.48 W cm^−2^, 3 min). Intracellular ROS generation was detected with DCFH-DA (green) and cell nuclei was stained with DAPI (blue). Scale bar: 50 μm. Figure S5. Flow cytometry analysis of apoptosis/necrosis of 4T1 cells incubated with pCy, free TPZ or TPZ@pCy (50 μg mL^−1^ of pCy) for 2 h and then irradiated with 808 nm (0.48 W cm^−2^, 3 min) or in dark, using the Annexin V-FITC/PI staining. Figure S6. Tumor weight of 4T1-bearing tumor mice received various treatments. Data are shown as mean ± S.D. (*n* = 6). * *p* < 0.05, ** *p* < 0.01. Figure S7. Body weight variation of tumor-bearing mice during treatment periods. Data are shown as mean ± S.D. (*n* = 6). Figure S8. Representative photographs of H&E staining of major organs (heart, liver, spleen and kidney). Scale bar: 100 μm.
