# Supplementary material for: Light-Decomposable Polymeric Micelles with Hypoxia-Enhanced Phototherapeutic Efficacy for Combating Metastatic Breast Cancer
Source: Pharmaceutics. 2022 Jan 21;14(2):253. doi: 10.3390/pharmaceutics14020253 (PMC8877442; doi:10.3390/pharmaceutics14020253)
Supplement: Supplementary file 1 [file pharmaceutics-14-00253-s001.zip › pharmaceutics-1492640-supplementary.pdf]

pharmaceutics

Supplementary Materials: Light-Decomposable Polymeric Micelles with Hypoxia-Enhanced Phototherapeutic Efficacy for Combating Metastatic Breast Cancer

Yuanyuan Li, Aiyang Tong, Peiyuan Niu, Wenjing Guo, Yangye Jin, Yi Hu, Pei Tao and Wenjun Miao

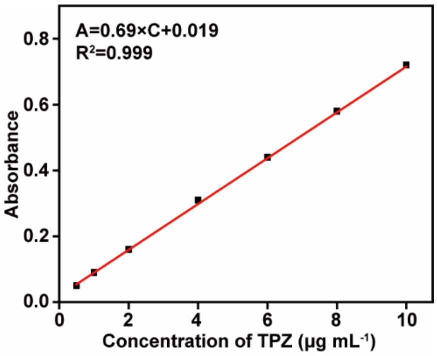

Figure S1. The calibration curve of TPZ in methanol, concentration range: 0.5-10 μg mL^{-1}.

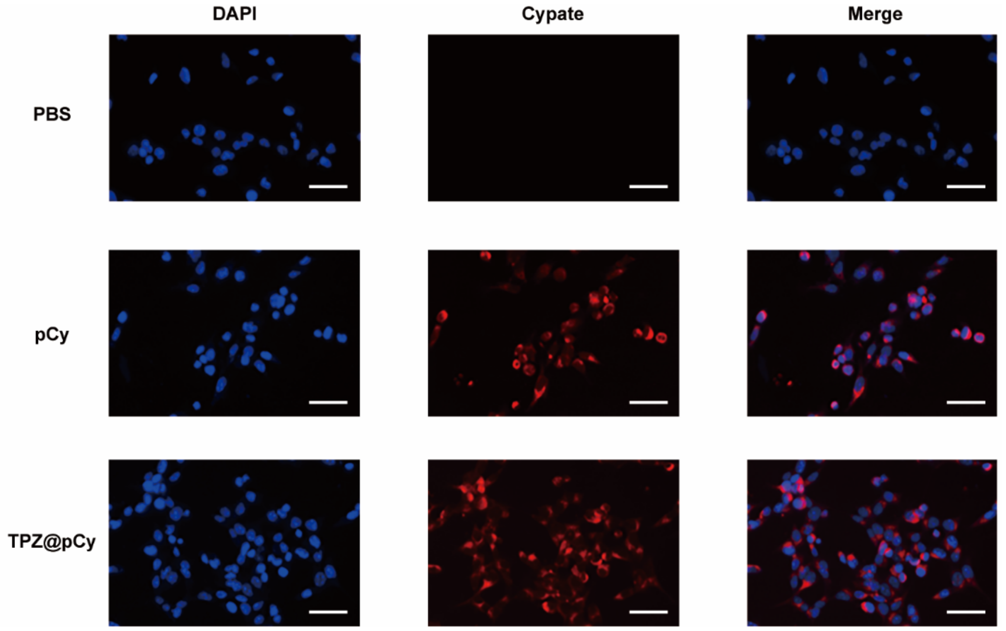

Figure S2. Fluorescence images of 4T1 cells treated with PBS, pCy or TPZ@pCy (20 μg mL^{-1}, equivalent to pCy) for 2 h, respectively. Cell nuclei was stained with DAPI (blue). Scale bar: 50 μm.

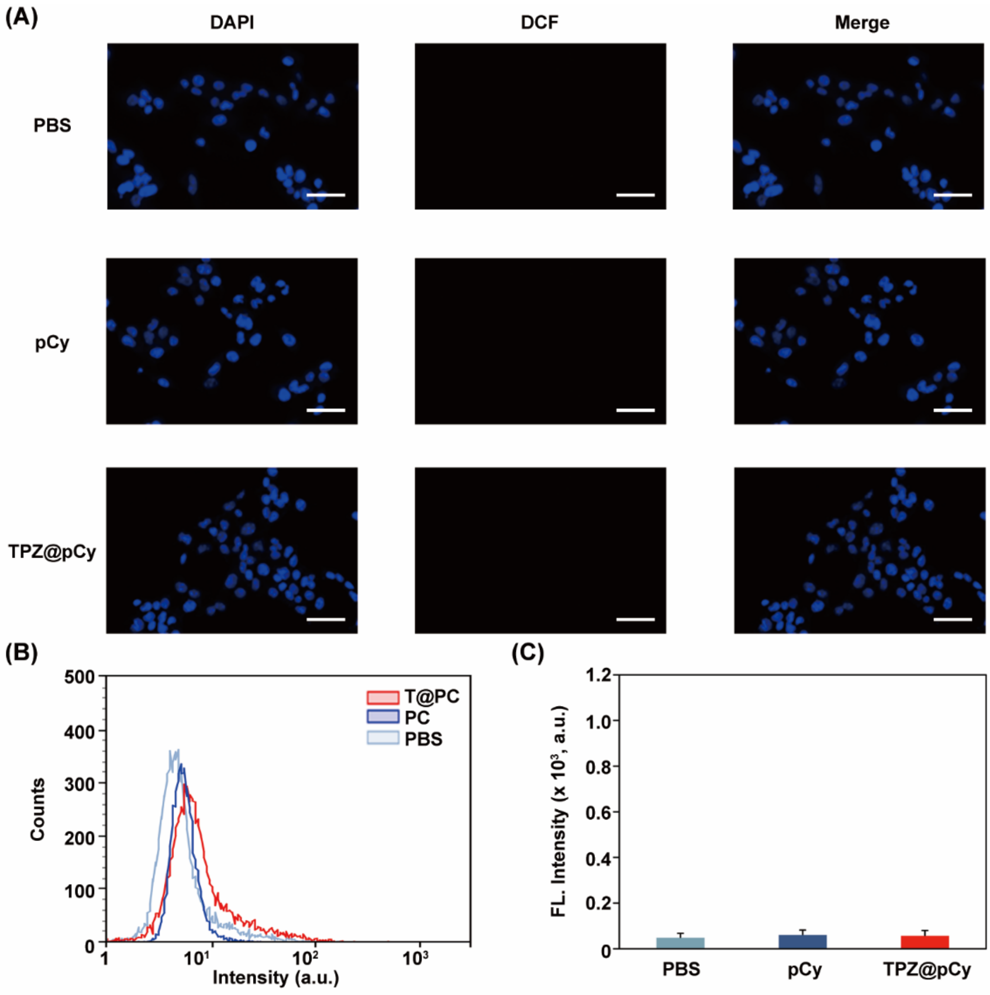

Figure S3. Fluorescence images of 4T1 cells incubated with PBS, pCy or TPZ@pCy (50 μg mL^{-1} of pCy) for 2 h in dark. (**A**) Intracellular ROS generation was detected with DCFH-DA (green) and cell nuclei was stained with DAPI (blue). Scale bar: 50 μm. (**B**) Flow cytometry analysis of 4T1 cells treated as identical conditions as above. (**C**) Plot showing the fluorescence intensity corresponding to (**B**).

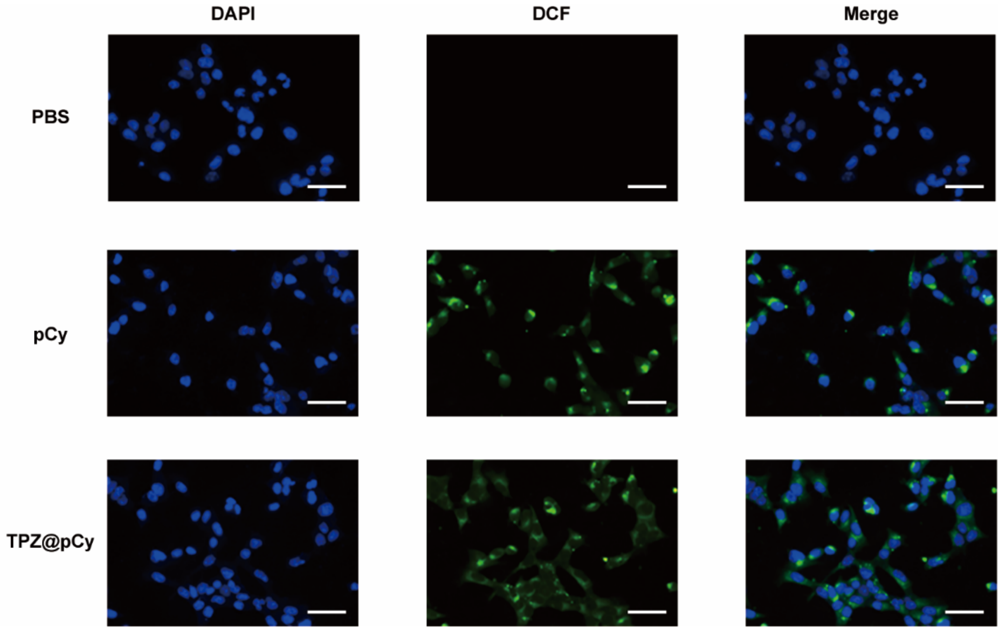

Figure S4. Fluorescence images of 4T1 cells incubated with PBS, pCy or TPZ@pCy (50 μg mL^{-1} of pCy) for 2 h and irradiated with 808 nm laser (0.48 W cm^{-2}, 3 min). Intracellular ROS generation was detected with DCFH-DA (green) and cell nuclei was stained with DAPI (blue). Scale bar: 50 μm.

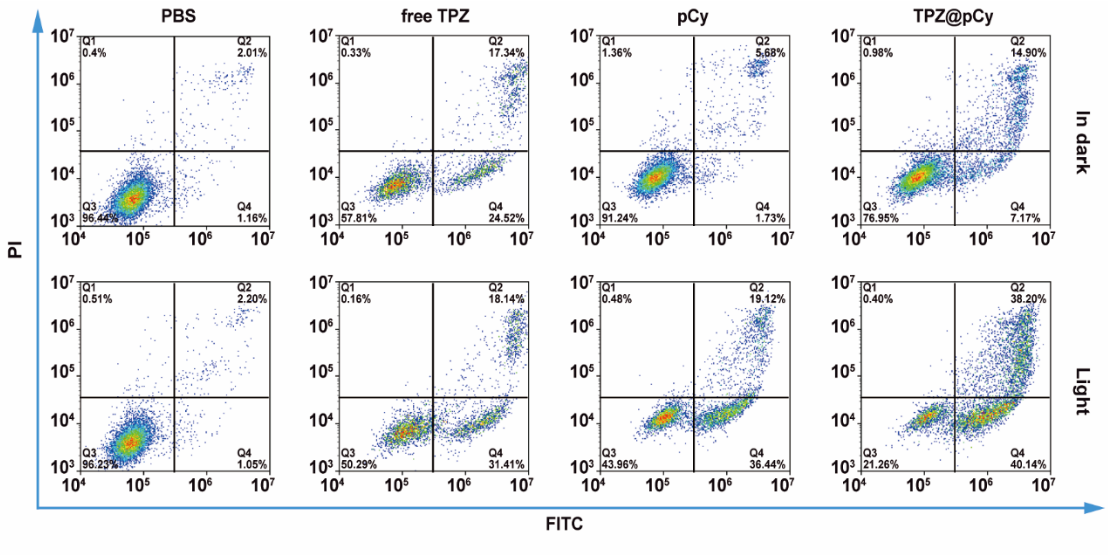

Figure S5. Flow cytometry analysis of apoptosis/necrosis of 4T1 cells incubated with pCy, free TPZ or TPZ@pCy (50 μg mL^{-1} of pCy) for 2 h and then irradiated with 808 nm (0.48 W cm^{-2}, 3 min) or in dark, using the Annexin V-FITC/PI staining.

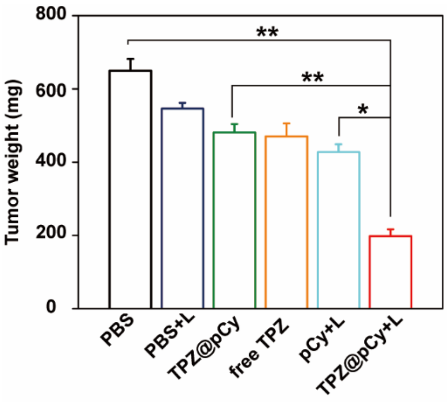

Figure S6. Tumor weight of 4T1-bearing tumor mice received various treatments. Data are shown as mean ± S.D. ($n = 6$). * $p < 0.05$, ** $p < 0.01$.

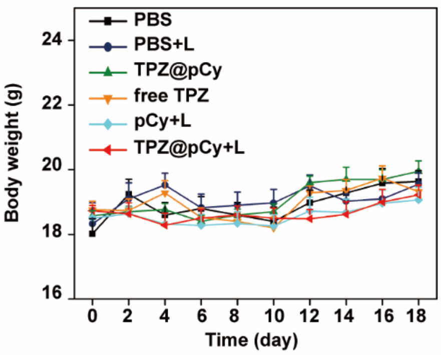

Figure S7. Body weight variation of tumor-bearing mice during treatment periods. Data are shown as mean ± S.D. ($n = 6$).

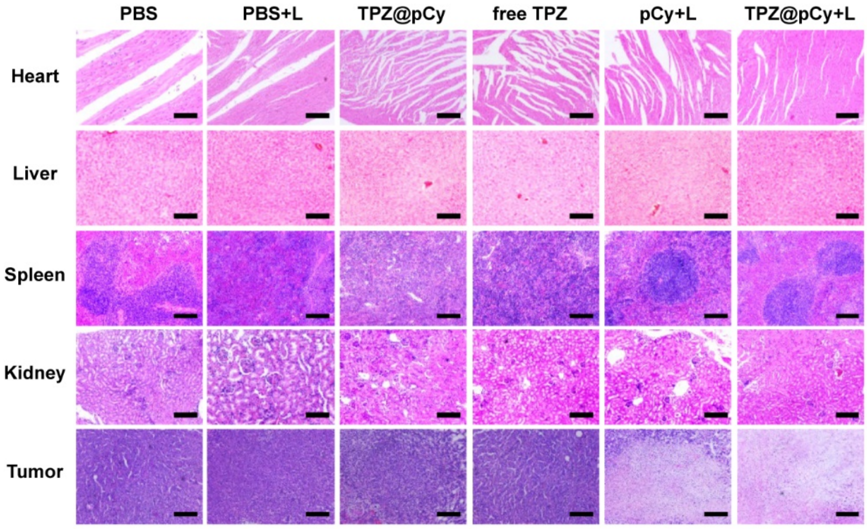

Figure S8. Representative photographs of H&E staining of major organs (heart, liver, spleen and kidney). Scale bar: 100 μm.
